# A Major Effect Gene Controlling Development and Pathogenicity in *Botrytis cinerea* Identified Through Genetic Analysis of Natural Mycelial Non-pathogenic Isolates

**DOI:** 10.3389/fpls.2021.663870

**Published:** 2021-04-14

**Authors:** Wilson Acosta Morel, Francisco Anta Fernández, Riccardo Baroncelli, Sioly Becerra, Michael R. Thon, Jan A. L. van Kan, José María Díaz-Mínguez, Ernesto Pérez Benito

**Affiliations:** ^1^Spanish-Portuguese Institute for Agricultural Research (CIALE), Department of Microbiology and Genetics, University of Salamanca, Salamanca, Spain; ^2^Laboratory of Phytopathology, Wageningen University, Wageningen, Netherlands

**Keywords:** gray mold, bulked segregant analysis, genetic complementation, DNA binding domain, acetyl transferase

## Abstract

*Botrytis cinerea* is a necrotrophic plant pathogenic fungus with a wide host range. Its natural populations are phenotypically and genetically very diverse. A survey of *B. cinerea* isolates causing gray mold in the vineyards of Castilla y León, Spain, was carried out and as a result eight non-pathogenic natural variants were identified. Phenotypically these isolates belong to two groups. The first group consists of seven isolates displaying a characteristic mycelial morphotype, which do not sporulate and is unable to produce sclerotia. The second group includes one isolate, which sporulates profusely and does not produce sclerotia. All of them are unresponsive to light. Crosses between a representative mycelial non-pathogenic isolate and a highly aggressive field isolate revealed that the phenotypic differences regarding pathogenicity, sporulation and production of sclerotia cosegregated in the progeny and are determined by a single genetic locus. By applying a bulked segregant analysis strategy based on the comparison of the two parental genomes the locus was mapped to a 110 kb region in chromosome 4. Subcloning and transformation experiments revealed that the polymorphism is an SNP affecting gene Bcin04g03490 in the reference genome of *B. cinerea*. Genetic complementation analysis and sequencing of the Bcin04g03490 alleles demonstrated that the mutations in the mycelial isolates are allelic and informed about the nature of the alterations causing the phenotypes observed. Integration of the allele of the pathogenic isolate into the non-pathogenic isolate fully restored the ability to infect, to sporulate and to produce sclerotia. Therefore, it is concluded that a major effect gene controlling differentiation and developmental processes as well as pathogenicity has been identified in *B. cinerea*. It encodes a protein with a GAL4-like Zn(II)2Cys6 binuclear cluster DNA binding domain and an acetyltransferase domain, suggesting a role in regulation of gene expression through a mechanism involving acetylation of specific substrates.

## Introduction

*Botrytis cinerea* is an endemic phytopathogenic fungus with a necrotrophic lifestyle (van Kan, 2006) and a very wide host range (Elad et al., 2016). It is the most important postharvest decay pathogen (Romanazzi and Feliziani, 2014) and the second most economically significant plant pathogen (Dean et al., 2012). It is suited with a large array of pathogenicity factors which enables the pathogen to use multiple strategies to infect many different species, essentially all aerial parts of the plant and both in the field and during storage (van Kan, 2006). In nature the fungus can reproduce both asexually and sexually. It produces different cell and tissue types, including mycelium, micro- and macroconidia, sclerotia, apothecia and ascospores, which enable its reproduction and survival under different conditions. Macroconidia, produced abundantly on colonized plant tissues, constitute its main dispersal structure. Sclerotia allow the fungus to survive in adverse environmental conditions, germinating to produce mycelium and macroconidia when favorable conditions are reestablished. The sclerotia are also functional during the sexual reproduction of the fungus, being fertilized by the microconidia provided by an isolate of the opposite sexual type. Fertilization gives rise to the development of fruiting bodies, the apothecia, where ascospores are produced, which can also be dispersed and infect host tissues. The arsenal of pathogenicity factors of the fungus, together with its versatility in reproduction and dispersal, make this pathogen difficult to control (Williamson et al., 2007).

Genetic analysis helps to draw the links between phenotype and genotype and to determine the genetic basis of traits. *B. cinerea* natural populations are known to display high phenotypic and genetic variation. Paul (1929) already distinguished three morphological types, sclerotial, sporulating and mycelial, based on the observation that field isolates produced predominantly sclerotia, conidia or mycelia. Several authors later described these morphotypes and even considered subtypes (Faretra et al., 1988; Martinez et al., 2003: Korolev et al., 2008). Variation in many diverse physiological aspects, vegetative growth, secondary metabolism, resistance to fungicides, response to light, and in virulence have also been extensively reported (Raposo et al., 1996; Kerssies et al., 1997; Martinez et al., 2003; Leroch et al., 2011; Schumacher et al., 2012; Canessa et al., 2013; Soltis et al., 2019). Undoubtedly, this phenotypic variation relies on the existing genetic variation in natural populations. The characterization of genetic variation, either natural or induced, and the analysis of segregating populations facilitates the construction of genetic maps as well as the functional analysis of genetic factors. On the other hand, quantification of genetic variability and evaluation of the mode in which this variation is structured provides valuable information about population structure and about the mode in which different factors, geography, host specialization, cultural practices, contribute to shape it (Walker et al., 2015). Understanding the basis of phenotypic variation has been facilitated by the development of molecular markers and recently by the affordability of genome sequencing methodologies.

The availability of the sequence of a reference genome offers a powerful tool for the genetic analysis of any biological system. First draft versions of the genome of two *B. cinerea* isolates, B05.10 and T4, were published in 2011 (Amselem et al., 2011). A gapless sequence of the isolate B05.10 (van Kan et al., 2017) has a total length of 42.6 Mb divided over 18 chromosomes, two of them (Chr17 and Chr18) very small. Community efforts to annotate this genome have provided precise gene models that facilitate functional studies and transcriptomic analyses in this species (Pedro et al., 2019). The characterization of the variation between sequenced genomes has facilitated the design of cloning strategies based on genetic mapping that have made possible the identification of the polymorphisms responsible for phenotypic variation in traits of interest (Schumacher et al., 2012; van Kan et al., 2017).

The sequence of reference genomes facilitates the assessment of variation at the genome scale in natural populations. Whole genome resequencing of populations of *B. cinerea* isolates have identified high levels of standing diversity and have shown it has a high level of recombination and genomic admixture (Atwell et al., 2015, 2018; Soltis et al., 2019). Genome-wide association studies for the analysis of virulence in *B. cinerea* on *Solanum lycopersicum* and *S. pimpinellifolium*, indicated that the genetic architecture of virulence is highly quantitative (Soltis et al., 2019). This provides support to the widely accepted notion that virulence in *B. cinerea* is polygenic and depends on the participation of an array of pathogenicity factors in the ability of the pathogen to infect the host plant. Interestingly, a pool of large-effect polymorphisms segregating in the populations were found (Atwell et al., 2018), among them the reported loss of function polymorphism for the VELVET gene (Schumacher et al., 2012). Therefore, natural populations are a useful source of variation in both major and minor effect genetic factors on phenotypes.

Bulked segregant analysis (BSA) is a QTL mapping technique devised to identify molecular markers tightly linked to genetic loci involved in the determination of a trait of interest (Magwene et al., 2011). The technique was initially developed to identify RFLP and RAPD markers linked to disease resistance genes in plants (Michelmore et al., 1991). It is more easily implemented for traits determined by single genes with major effect that condition clear and contrasting phenotypes, but it can also be used for complex traits (Magwene et al., 2011). It involves the generation of a segregating population from a genetic cross in which the individuals are assayed for the trait of interest. Two bulked DNA samples are generated from progenies with contrasting phenotypes and genotyped with molecular markers polymorphic between the parental lines. Allelic frequencies should be very similar in the two bulks in regions without loci affecting the trait. For regions containing causal loci, the allelic frequencies of associated markers should exhibit differences between bulks, becoming more significant as the linkage relationships are closer. The resolution of this kind of methodology depends on the number of markers analyzed simultaneously and on the precision in the estimation of the allelic frequencies, which is a function of the number of individuals analyzed in each of the progeny groups (Magwene et al., 2011; Swinnen et al., 2012; Schlötterer et al., 2014). Sequencing the genomes of the parental lines and determining the polymorphisms between them greatly enhance the power of BSA. *In Saccharomyces cerevisiae*, NGS-assisted BSA has been successfully applied to uncover the genetic basis of Mendelian traits (Birkeland et al., 2010; Wenger et al., 2010) and multi-gene traits (Ehrenreich et al., 2010; Magwene et al., 2011; Parts et al., 2011; Swinnen et al., 2012). In *Neurospora crassa* the methodology has revealed that the temperature sensitive cell cycle mutation ndc-1 is allelic with the gene for Ornithine decarboxylase (Pomraning et al., 2011).

Transcription factors (TFs) play key roles in the regulation of gene expression by binding to DNA in a sequence-specific manner (Weirauch and Hughes, 2011). They represent the link between the perception and transmission of the signal and the target genes expression. TFs are typically classified by their DNA-binding motif. Representants of 80 TF families are typically found in fungal genomes (Shelest, 2017). The largest family of fungal TFs is the zinc cluster (C6 zinc finger) family (Shelest, 2008). Members of this multifunctional family control several crucial fungal processes including sugar metabolism, gluconeogenesis and respiration, amino acid metabolism and vitamin synthesis, mitosis, meiosis, chromatin remodeling, nitrogen utilization, peroxisome proliferation, stress response and multidrug resistance (reviewed by MacPherson et al., 2006). Like most transcription factors, zinc cluster proteins contain several functional domains. The DNA binding-domain of this family consists of six cysteine residues, arranged in the highly conserved motif CX2CX6CX6CX2CX6C, that bind two zinc atoms. The zinc clusters can interact with DNA as monomers or as homo- or hetero-dimers. It was first described and characterized in the *S. cerevisiae* Gal4 protein, a transcriptional activator that regulates galactose utilization (Pan and Coleman, 1990). The cysteine-rich DNA binding domain is commonly located at the N terminus. The regulatory domain separates the DNA binding domain and the activator domain. It is less conserved and not always present in members of the family. Most often C-terminally located, the acidic domain acts as activation domain. It is not a conserved domain and its function and structure is varied in the family (reviewed by MacPherson et al., 2006). 222 TFs of this class were annotated in the genome of the B05.10 isolate (Amselem et al., 2011). The Zn2Cys6 TF BcGaaR has been shown to be required for induction of Gal-inducible genes and growth of *B. cinerea* on Galacturonic acid (Zhang et al., 2015). BcBot6 has been found to be a major positive regulator of Botrydial biosynthesis (Porquier et al., 2016).

We are interested in the characterization of the local populations of *B. cinerea* in the vineyards of Castilla y León (Spain). In the course of the analysis of field isolates from different geographic origins large differences in virulence were observed among isolates (Acosta Morel et al., 2019). In this work, we extend our collection of natural isolates altered in pathogenicity and perform a genetic and genomic analysis of their differences. A novel major effect gene controlling development processes and pathogenicity is described.

## Materials and Methods

### Organisms and Growth Conditions

The *B. cinerea* isolates used in this work are indicated in Table 1. They form part of the collection of field isolates maintained by the CIALE (University of Salamanca, Spain). They were sampled and purified as described previously (Acosta Morel et al., 2019). *B. cinerea* isolates were routinely grown on MEA plates and incubated at 22°C under permanent darkness or permanent light conditions or under a 16 h photoperiod (light generated by Cool White Osram L 36W/840 fluorescent bulbs).

**TABLE 1:** Isolates used in this work.

					Ability to infect leaves					
Isolate	Origin	Host	Collection Date	References	*V. vinifera*	*P. vulgaris*	Sporulation	Sclerotia formation	Response to light	Mating Type
B05.10	Unknown	Germany		Büttner et al (1994)	Yes	Yes	Yes	Yes	Yes	MAT 1-1
B116	Rueda (Spain)	*V. vinifera* (Tempranillo)	10/08/2002	Acosta Morel et al. (2019)	No	Yes	Yes	No	No	MAT 1-2
B217	San Rom n de Hornija (Spain)	*V. vinifera* (Tempranillo)	8/28/2007	Acosta Morel et al. (2019)	No	No	No	No	No	MAT 1-2
B286	Aldead vila de la Ribera (Spain)	*V. vinifera* (Juan Garc a)	10/09/2007	This work	No	No	No	No	No	ND
B350	Aranda de Duero (Spain)	*V. vinifera* (Tempranillo)	10/08/2007	This work	No	No	No	No	No	ND
B371	Aranda de Duero (Spain)	*V. vinifera* (Tempranillo)	10/08/2007	Acosta Morel et al. (2019)	No	No	No	No	No	MAT 1-2
B448	Roa (Spain)	*V. vinifera* (Garnacha)	10/08/2007	Acosta Morel et al. (2019)	Yes	Yes	Yes	Yes	Yes	MAT 1-1
B459	Aranda de Duero (Spain)	*V. vinifera* (Tempranillo)	10/08/2007	Acosta Morel et al. (2019)	No	No	No	No	No	MAT 1-2
B468	Aranda de Duero (Spain)	*V. vinifera* (Tempranillo)	10/08/2007	This work	No	No	No	No	No	ND
B471	Aranda de Duero (Spain)	*V. vinifera* (Tempranillo)	10/08/2007	Acosta Morel et al. (2019)	No	No	No	No	No	MAT 1-1

*The phenotypes in relation to their ability to infect leaves of two host species, to sporulate, to produce sclerotia and to respond to light are summarized. The mating type is indicated (ND: not determined).*

Bean plants (*Phaseolus vulgaris*) cv Blanca Riñon were grown in natural substrate for 2 weeks in the green house under a 16 h photoperiod. *Vitis vinifera* plants variety Tempranillo were maintained in the green house in the same conditions.

### Standard Molecular Methods

Genomic DNA was obtained from mycelium cultured in liquid Gamborg’s B5 salts medium (AppliChem) supplemented with 10 mM sucrose and 10 mM KH_2_PO_4_ (pH 6.0). DNA to be sequenced with the Ion Torrent sequencer was purified using the protocol described by Raeder and Broda (1985). Estimations of DNA concentrations were obtained using the Qubit dsDNA HS (High Sensitivity) Assay Kit (Invitrogen, Thermofisher) with the Qubit Fluorometer.

Routine PCR reactions were carried out using the Taq Polymerase from Biotools. The amplifications of the Bcin04g03490 alleles were performed using the KAPA HiFi HotStart DNA polymerase (Roche) following manufacturers recommendations. The oligonucleotides used in this work are presented in Supplementary Table 1.

Ligations of fragments generated by restriction enzymes were done with the T4 DNA ligase (Roche). Cloning of genomic DNA fragments in plasmid pWAM6 were carried out using the Gateway BP Clonase II Enzyme Mix according to manufacturer’s indications (Thermofisher). Plasmid pWAM6 was derived from plasmid pNR4 (Zhang et al., 2011) by replacing the 1,9 kb *Xba*I-*Hin*dIII fragment containing the nourseothricin resistance cassette with a 2,6 kb *Xba*I-*Hin*dIII fragment containing the hygromycin resistance cassette from pOHT (Hilber et al., 1994).

Plasmids and PCR fragments were sequenced at the Sequencing Service of NUCLEUS, University of Salamanca, with an ABI PRISM 377 automatic sequencer. Sequences were handled using Geneious R.11 (Biomatters, Auckland, New Zealand).

### *B. cinerea* Transformation

*B. cinerea* protoplasts were transformed following the protocol described by ten Have et al. (1998) with the modifications indicated by Reis et al. (2005). When the strain to be transformed did not produce spores, protoplasts were generated from mycelium derived from small agar plugs with actively growing mycelium inoculated and cultivated for 72 h in ME medium.

### Genetic Complementation Analysis

The Bcin04g03490 alleles from three mycelial non-pathogenic isolates, B217, B459 and B471, together with the allele from the B371 isolate, were amplified as a 3.489 nt fragment which includes the entire gene coding region plus 588 nt of its 5′ upstream sequence and 586 nt from its 3′ downstream sequence with oligonucleotides 4S7-F and 4S7-R-1 and cloned in pWAM6 giving rise to plasmids pCAa217, pCAa459, pCAa471 and pCAa371, respectively. The allele from the sporulating, non-pathogenic isolate B116 was also amplified and cloned, generating plasmid pCAa116. Plasmid pBAS23, which contains the a448 allele, was used as a reference control. All these plasmids were transformed into B371 protoplasts. Primary transformants were selected and transferred to fresh selective plates. Sporulation and production of sclerotia were evaluated in the transformants generated as indicators of functional complementation.

### Ion Proton Sequencing and Determination of Polymorphisms

Genomic DNA from the B371 and B448 isolates, as well as the two genomic DNA pools generated from the progeny in cross B371 x B448, were sequenced using the Ion Proton (Life Technologies) sequencer by Bioarray (Alicante, Spain). Reads (mean length 163-180 nt) were mapped on the ASM15095 v229 *Botrytis cinerea* B05.10 assembly^1^ using the Torrent Mapping Alignment Software (TMAP). Average coverage was 100× (with genome base coverage at 20× higher than 95% in all the samples). Torrent Variant Caller (TVC) software was used to obtain sequence variants and their frequencies, and SnpEff v4.2 software was implemented to annotate these variants and the impact of mutations in coding sequences.

### BSA

For the BSA analysis the list of polymorphisms generated with TVC were used. Heterozygous calls in the B371 and B448 genomes were filtered and a list of polymorphisms exclusive of each parental isolate was generated. Within the progeny two groups of individuals were selected: the first one, group A, consisted of 50 individuals resembling parental isolate B448, and the second, group B, consisted of 50 individuals resembling parental isolate B371. Genomic DNA was extracted from each single isolate and for each of the two groups, equal amounts of DNA from each of the 50 individuals were combined. The two pools of genomic DNA were sequenced and the frequencies in each DNA pool of the polymorphisms specific of either isolate B448 or of isolate B371 were determined. For the association mapping analysis, only high-quality SNPs (deep coverage > 20 and quality score > 500) were selected. The distribution of the polymorphisms specific of one or the other parental isolate in the two progeny groups was analyzed by calculating the difference in the frequency of each polymorphism in DNA pool B and in DNA pool A (f”x” B – f”x” A). This SNP index (*Y* axis) was represented along the coordinates of each chromosome (*X* axis) in the B05.10 reference genome. For markers not linked to the locus involved in the determination of the phenotypic difference, the allelic frequencies should be similar in both pools and then the SNP index plot for those chromosomic regions should vary slightly around the “0” value. For markers associated with the locus of interest, allelic frequencies would differ in both DNA pools, being the differences larger as the markers are more closely linked to it. The SNP index would reach maximal values (close to + 1) for B371 exclusive alleles only present in the non-aggressive progeny DNA pool and minimal values (close to -1) for B448 exclusive alleles only present in the aggressive progeny DNA pool.

### Crosses and Gene Mapping

Sexual crosses were performed following the protocol of Faretra et al. (1988). Mature apothecia were sampled and crushed in water to release the ascospores. The spore suspension was filtered through glasswool and plated at low density on MEA plates. Single ascospore germlings were transferred 24 h later to fresh MEA plates for visual inspection and propagation.

### Inoculations and Virulence Assays

Inoculations and evaluation of differences in virulence in *V. vinifera* variety Tempranillo leaves were carried out using a mycelium plug based inoculation system in non-wounded leaves as described previously (Acosta Morel et al., 2019). The ability to infect *P. vulgaris* leaves was analyzed using whole plants. In both systems, isolates that did not expand from the inoculation site were considered to be unable to cause any lesion.

### Construction of Bcin04g03490 Mutants in B05.10

A gene replacement cassette was built up using plasmid pMAS25, generated to replace gene *Bcmimp*1 (Benito-Pescador et al., 2016), as a backbone. First the *Kpn*I-*Hin*dIII fragment containing the 5′ upstream region of gene *Bcmimp*1 in plasmid pMAS25 was replaced by a 606 nt *Kpn*I-*Hin*dIII fragment amplified from genomic DNA using oligonucleotides 03490-5′*Kpn*I and 03490-5′*Hind*III. From the resulting plasmid, the *Xba*I-*Not*I fragment containing the 3′ downstream region of gene *Bcmimp*1 was replaced by a 753 nt *Xba*I-*Not*I fragment amplified from genomic DNA using oligonucleotides 03490-3′*Xba*I and 03490-3′*Not*I. The plasmid generated was used to amplify the replacement cassette with the external primers flanking the construct. The PCR product was used to transform *B. cinerea* B05.10. Primary transformants were transferred to fresh selection plates. Enrichment in transformed nuclei was pursued by making successive replicas (6) to fresh selection plates. By visual inspection, several transformants displaying the non-sporulating phenotype were identified. Two transformants that have resulted from gene replacement by double homologous recombination were confirmed by PCR using diagnostic primer combinations (Figure 7).

### Evolutionary Analyses

Selected orthologous of Bcin04g03490 encoded protein were identified through a BLAST approach, retrieved from GenBank and aligned with MAFFT v7.450 (Katoh and Standley, 2013). Best substitution model and its parameter values were selected using ProtTest v3.4.2 (Darriba et al., 2011). Phylogenetic tree was reconstructed using Bayesian MCMC analysis (MrBayes v3.2.7; Ronquist and Huelsenbeck, 2003) constructed from the alignment under the WAG + I evolutionary model and the gamma distribution calculated using four rate categories and homogeneous rates across the tree. The posterior probabilities threshold was selected as over 50%.

## Results

### Phenotypic Characterization of *B. cinerea* Non-pathogenic Natural Variants

**FIGURE 1:**
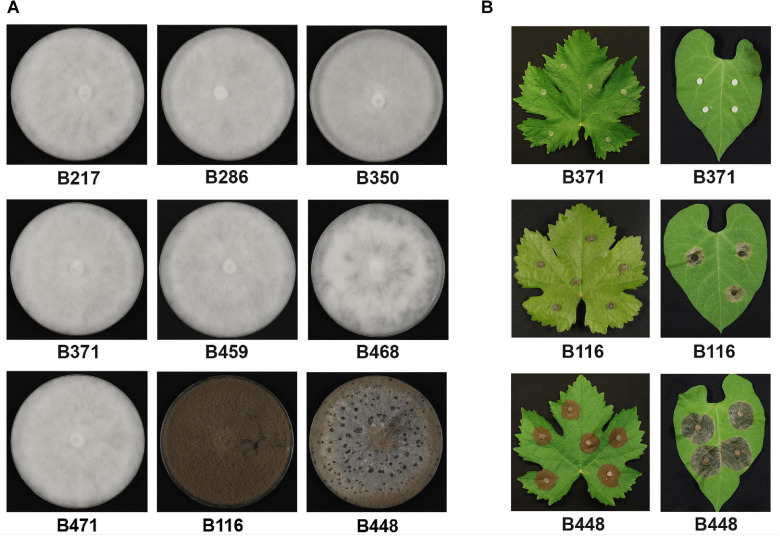
Phenotypic characterization of the non-pathogenic field isolates analyzed in this work. **(A)** Growth in MEA plates 14 days after inoculation in dark conditions. **(B)** Pathogenicity assays on *V. vinifera* and on *P. vulgaris* leaves. Non-wounded leaves were inoculated with the indicated isolates (B371 is representative of the seven mycelial isolates) and incubated for 72 h at 22°C with a 16 h photoperiod.

We have previously identified four *B. cinerea* isolates, B217, B371, B459, and B471, unable to infect *V. vinifera* leaves and displaying a mycelial morphotype. In addition, a fifth isolate non-pathogenic on *V. vinifera* leaves was purified which sporulated profusely, B116 (Acosta Morel et al., 2019). In order to increase the number of natural isolates altered in pathogenicity, we extended the physiological evaluation of our field isolates collection with 170 additional isolates. When inoculated on *V. vinifera* leaves, isolates B286, B350 and B468 were unable to cause lesions. These three isolates all displayed the mycelial morphotype characteristic of isolates B217, B371, B459 and B471 (Figure 1). The 7 mycelial isolates non-pathogenic on *V. vinifera* leaves were also unable to infect *P. vulgaris* leaves. Isolate B116 did infect bean leaves, although it caused much smaller lesions in this host than the aggressive field isolate B448 (Acosta Morel et al., 2019) (Figure 1). This B448 isolate sporulates and produces numerous sclerotia when cultured in synthetic solid media in dark conditions and resembles very much the phenotype of the reference isolate B05.10. Interestingly, none of the 8 non-pathogenic isolates produced sclerotia on MEA plates or on PDA plates. Therefore, among the non-pathogenic isolates identified in this analysis two groups can be distinguished: a first class consists of seven isolates of the mycelial morphotype, which do not sporulate, do not produce sclerotia and neither infect *V. vinifera* nor *P. vulgaris* leaves, and a second class represented by a single isolate, B116, which sporulates, does not produce sclerotia, is unable to infect *V. vinifera* leaves but, although being less aggressive than the B448 isolate, does infect *P. vulgaris* leaves. In our sampling, it has been shown that multiple infections of the same bunch by genetically different individuals occur frequently (Acosta Morel et al., 2019). It is interesting to note that tracing the origin of the non-aggressive isolates indicated that they were purified from symptomatic bunches from which other aggressive *B. cinerea* isolates were also purified.

### The Non-pathogenic Natural Mutants Are Blind

**FIGURE 2:**
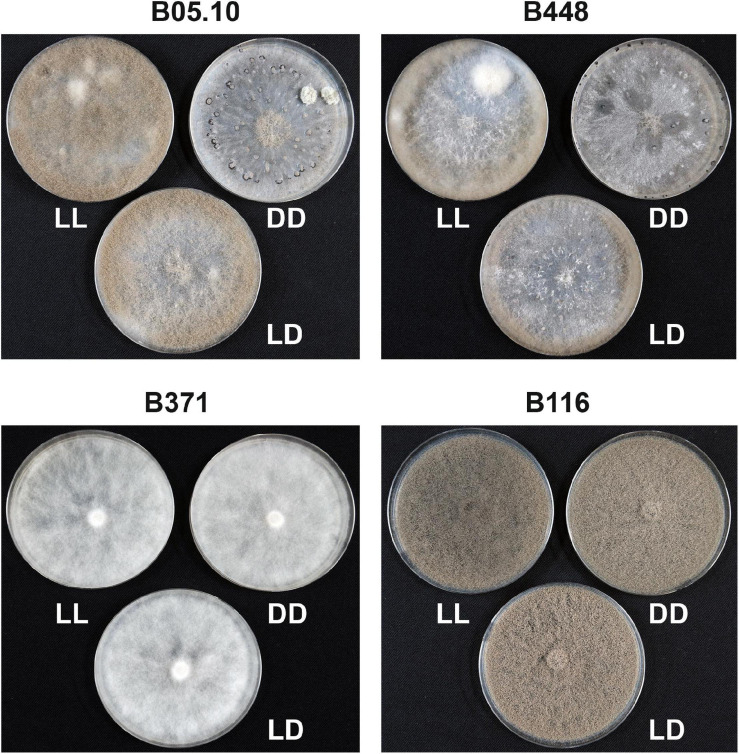
Evaluation of the effect of light on sporulation and sclerotia production in the non-pathogenic natural mutants identified in comparison with the aggressive field isolate B448 and the reference isolate B05.10. Isolates were grown for 14 days in permanent light (LL), constant darkness (DD) or a 16 h photoperiod (LD).

As none of the non-pathogenic mutants produced sclerotia in conditions in which they should, their response to light was evaluated. As shown in Figure 2, the aggressive isolate B448 behaves as the reference isolate B05.10. Sporulation was stimulated in light conditions (either continuous or discontinuous) while sclerotia were produced in permanent dark conditions. Mycelial isolate B371 neither produced spores nor sclerotia in any light regime. B116 sporulated profusely independent of the light conditions and never produced sclerotia. Therefore, all the non-pathogenic natural mutants do not respond to light and behave as blind isolates.

### Genetic Analysis of the Mycelial Non-pathogenic Isolates

As clear and contrasting phenotypes were identified among the collection of field isolates in the ability to infect, sporulate and produce sclerotia, the genetic analysis of these traits could be considered. We decided to first initiate the characterization of the seven mycelial isolates by analyzing the segregation of the three traits in crosses carried out between selected representative mycelial non-aggressive isolates and our reference aggressive field isolate B448. This isolate carries the MAT 1-1 mating allele. Therefore, mycelial isolates carrying the MAT 1-2 mating allele, B371 and B459, were selected for crossing. The mycelial isolates do not produce sclerotia at all, but they produced microconidia. Crosses could be established using isolate B448 as the female parental strain. In cross B448 x B371 an offspring consisting of 183 single ascospore isolates was collected. The three traits under consideration were scored in the full set of descendants. 83 individuals displayed the combination of phenotypic alternatives characteristic of isolate B371, while 100 individuals showed the combination of phenotypes shown by isolate B448. This situation is indicative of a strict co-segregation of the three traits being analyzed. The proportion of the two classes, 83:100, informs about a 1:1 segregation. Therefore, it can be concluded that the three traits considered are under the control of a single genetic locus which has been altered in isolate B371. Interestingly, when the aggressiveness of the pathogenic progeny was quantified, large differences among individuals were observed (Supplementary Figure 1). This observation suggests that the wild type allele in isolate B448 of that major effect gene controlling sporulation, production of sclerotia and pathogenicity regulates the function of a number of pathogenicity genes which segregate in the aggressive progeny.

In the cross B448 x B459, an offspring of 82 individuals was generated. 37 of them were phenotypically like the B459 parental isolate and 45 were like the parental isolate B448 (not shown). These phenotypic proportions are also indicative of a 1: 1 segregation. The data obtained suggest that the phenotypic differences observed between isolates B448 and B459 are also due to the segregation of a single genetic locus.

### Mapping the Gene Altered in B371 by BSA

The cross B448 x B371 was selected for genetic and genomic analysis. In order to gain information about the sequence polymorphisms between these isolates both genomes were sequenced. High quality short reads were aligned to the B05.10 genome reference sequence. In total, 90.189 B371 isolate exclusive polymorphisms and 91.067 B448 isolate exclusive polymorphisms were identified in comparison with the B05.10 reference isolate genome. In addition, both isolates were found to share 165.690 polymorphisms in comparison with the reference genome.

**FIGURE 3:**
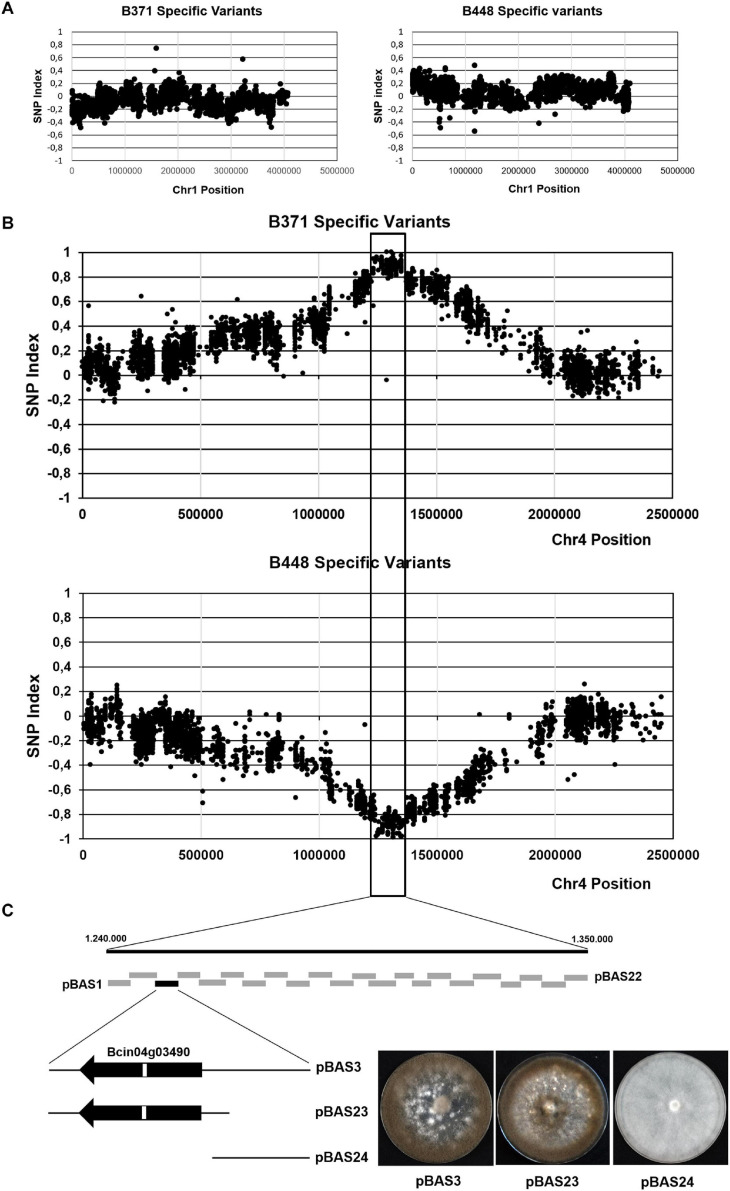
Mapping the major effect gene controlling development and pathogenicity in cross B448 x B371 by BSA. Plots of the distribution of frequencies of the polymorphisms specific of isolate B371 or specific of isolate B448 in Chr1 **(A)** and in Chr4 **(B)**. In each plot the *x*-axis represents the coordinates of the B05.10 isolate chromosome, used as the reference. The *y*-axis represents the SNP index value of each polymorphism. The vertical lines mark the region where the SNP index reaches values close to (+ 1) for B371 specific variants and close to (-1) for B448 specific variants. **(C)** Fine mapping by functional complementation. Boxes represent DNA fragments derived from the parental isolate B448 Chr4 in the region delimitated by BSA subcloned in plasmid pWAM6. The black box represents the clone which restored the wild type phenotype upon transformation of isolate B371. The fragment in pBAS3 was further subcloned in plasmids pBAS23 and pBAS24 (see text). The images show representative transformants obtained with the indicated plasmids cultured during 14 days in MEA plates at 22°C and dark conditions.

To identify the gene (or genes) altered in B371, a strategy based on BSA was devised. This approach should make it possible to identify polymorphisms specific of the B371 isolate co-segregating with the “non-pathogenic” phenotypic alternative and polymorphisms specific of the B448 isolate co-segregating with the “pathogenic” phenotypic alternative. Once the lists of polymorphisms between isolates B371 and B448 were available, the distribution of the frequencies of markers specific of each parental isolate was analyzed in two pools, A and B, of descendants from the B371 x B448 cross. Each pool consisted of 50 individuals: the first pool contained isolates resembling the pathogenic parental isolate B448 and the second pool contained isolates resembling the non-pathogenic parental isolate B371. For most chromosomes, the plots of the SNP index, oscillate close to “0” along the entire chromosome as shown for Chr1 (Figure 3A; plots for other chromosomes not shown). Chr4 was the only exception: in this case the two SNP plots peaked around coordinate 1.300.000, reaching maximal values for the one corresponding to the B371 isolate specific polymorphisms and minimal values for the one corresponding to the B448 isolate specific polymorphisms. Both plots delimitated a genomic region of markers associated with the segregating phenotypes of about 110 kb (between coordinates 1.240.000 and 1.350.000) (Figure 3B). This region includes 32 annotated genes in the genome of the reference strain B05.10.

### Functional Identification of the Gene Altered in B371

**FIGURE 4:**
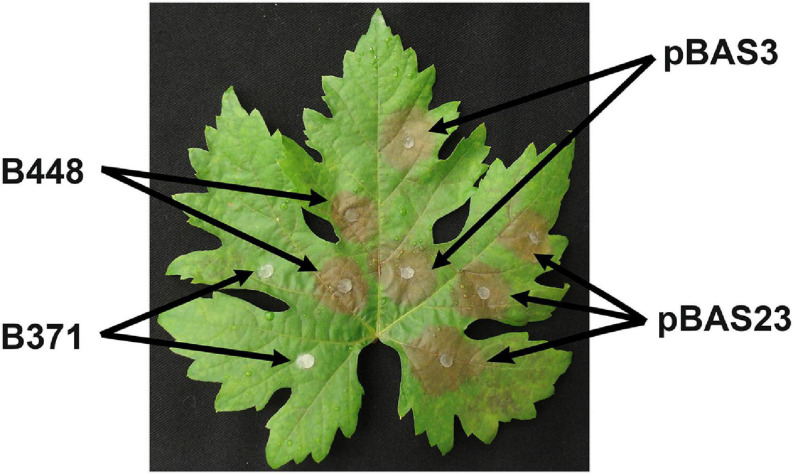
Pathogenicity in *V. vinifera* leaves of transformants obtained with plasmids pBAS3 and pBAS23 in comparison with isolates B448 and B371. Non-wounded leaves were inoculated with the indicated isolates and incubated at 22°C under a 16 h photoperiod. Image was taken 72 h after inoculation.

To narrow down the location of the gene, or genes, altered in isolate B371 the corresponding genomic region from isolate B448 was subcloned in fragments of about 5 kb in size amplified by PCR. The resulting plasmids were independently introduced by PEG mediated transformation in isolate B371 protoplasts. Primary transformants were transferred to selection plates and then visually inspected checking their ability to sporulate. With plasmid pBAS3 sporulating transformants were obtained (Figure 3C). These transformants were able to infect *V. vinifera* leaves (Figure 4).

**FIGURE 5:**
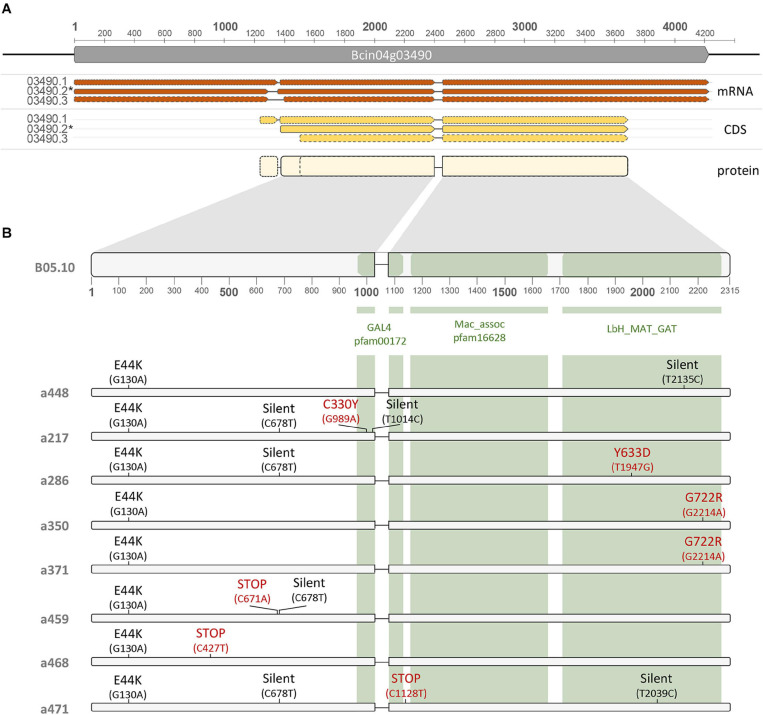
**(A)** Organization of the Bcin04g03490 locus and polymorphisms in the alleles from the mycelial non-pathogenic field isolates. The three splice variants annotated in the locus, the corresponding coding sequences and the predicted sizes of the encoded isoforms are presented. Continuous lines frame the representations of the Bcin04g03490.2 splice variant, CDS and encoded protein, considered as the references (*) in this study. **(B)** Regions in the Bcin04g03490.2 CDS encoding the GAL4, Mac_assoc and LbH_MAT_GAT functional domains, represented by green boxes, identified in the encoded protein. Below the B05.10 allele scheme, the polymorphisms identified in the Bcin04g03490 alleles of the mycelial field isolates and of isolate B448 are presented. For each allele, the nucleotide polymorphisms are indicated in parenthesis relative to the ATG codon. Above each polymorphism, the effect on the encoded protein is indicated. GenBank accession numbers of Bcin04g03490 alleles are the following: a448 – MW682868; a371 – MW682869; a217 – MW682870; a286 – MW682871; a350 – MW682872; a459 – MW682873; a468 – MW682874; a471 – MW682875.

The DNA fragment cloned in pBAS3 is 4.973 nt in size covering the sequence between nt positions 1.251.002 and 1.256.074. It contains a single gene, annotated in the B05.10 genome as Bcin04g03490 in the reverse strand and consisting of three exons separated by two introns (GeneID:36394109). Three splice variants are annotated for this gene (Figure 5A). Manual curation of the annotations provides limited support for splice variant Bcin04g03490.1 This would consider a first small (16 nucleotides) intron between positions 1.253.989 and 1.253.974 and a second intron of 50 nucleotides between positions 1.252.942 and 1.252.893. Translation of the predicted ORF from a start codon located at position 1.254.108 would generate a 794 amino acids protein (XM_024692527.1). However, detailed analysis of RNAseq data does not provide solid evidence for the processing of the first small intron predicted (data not shown). Splice variant Bcin04g03490.2 is derived from the consideration of a first intron, 63 nt in length, located in the 5′-UTR region, between positions 1.254.053 and 1.253.991, and the 50 nt long intron described for the first splice variant. Translation from the start codon at position 1.253.972 would result in a 754 amino acids protein (XP_024548305.1) (Figure 5A). Splice variant Bcin04g03490.3 is consequence of the utilization of an alternative 3′ splice junction for the first intron described in variant 2, located at position 1.253.948. Removal of this 106 nt long intron would condition the utilization of a downstream in frame translation start codon at position 1.253.840 and a 710 amino acids protein (XP_024548306.1) would be generated. Therefore, the three predicted proteins would share the last 710 aa with a 44, for Bcin04g03490P2, and a 84, for Bcin04g03490P1, amino acids extension at the amino terminus. In our analysis and descriptions, the gene model and splice variant originating the 754 amino acids protein will be considered as the reference and numbering will be given according to this model.

The DNA fragment in plasmid pBAS3 was subcloned in two plasmids: pBAS23, which contained gene Bcin04g03490 and its flanking sequences, 588 nt of the 5′ upstream region and 586 nt of the 3′ downstream region, amplified with oligonucleotides 4S7-F/4S7-R-1 (insert size 3.489 nt), and pBAS24, which contained the 1.956 nt fragment amplified with oligonucleotides 4S7-F-1/4S7-R and located 5′ upstream of Bcin04g03490 (which partially overlaps the insert in pBAS23). Both plasmids were transformed into B371 protoplasts. Only plasmid pBAS23 rescued the sporulation phenotype characteristic of the B448 isolate (Figure 3C). pBAS23 transformants were also able to infect *V. vinifera* leaves (Figure 4).

We then analyzed the polymorphisms in the 3.460 nt long DNA fragment from isolate B448 cloned in pBAS3 fragment and the sequence from the equivalent fragment from isolate B371 in comparison with the sequence of the reference isolate B05.10 (Table 2). Only one B371 specific SNP was identified, a C(G) to T(A) transition at position 1.251.759 which determines a G to R substitution at amino acid position 722 in the encoded protein, anticipated to have a moderate impact in the function of the protein (Figure 5B and Table 2). One B448 specific polymorphism was also identified, a A(T) to G(C) transition at position 1.251.838, this being a silent mutation. In addition, both isolates shared a polymorphism in relation to the B05.10 allele, a C(G) to T(A) transition at position 1.253.843, which determines a E to K substitution. Therefore, the C(G) to T(A) transition at position 1.251.759 affecting gene Bcin04g03490 is responsible for the alterations in development and pathogenicity that isolate B371 displays.

**TABLE 2:** Polymorphisms identified in the sequences of the B448 and B371 isolates in comparison with the sequence of the reference isolate B05.10 in the region cloned in plasmid pBAS3 (between nucleotide positions 1.251.101 and 1.254.541 from Chr4).

Position	Type of mutation	Nt change	Genome		Gen	Region	aa change	Impact
1251759	SNP	C(G) to T(A)		B371	Bcin04g03490	CR	G722R	MODERATE
1251838	SNP	A(T) to G(C)	B448		Bcin04g03490	CR	I695I	LOW
1253843	SNP	C(G) to T(A)	B448	B371	Bcin04g03490	CR	E44K	MODERATE

### The Protein Encoded by Bcin04g03490

Splice variant Bcin04g03490.2 is predicted to encode a hypothetical protein of 754 amino acids. Analysis of conserved domains identifies a GAL4-like Zn(II)2Cys(6) binuclear cluster DNA binding domain between residues 322 and 360, in the central part of the protein (Figure 5B). Between positions 554 and 745 a “LbM_MAT_GAT” region is found, characteristic of Maltose O-acetyltransferase (MAT) and Galactoside O-acetyltransferases (GAT). Between these two domains (positions 370 to 535) a “Mac-assoc” region, is found. This is described as a region of unstructured residues on fungal maltose acetyltransferase proteins linking the acetyltransferase domain and the Zn(II)2Cys(6) binuclear cluster. This organization of domains is characteristic of the domain architecture 11251539. On the basis of the conserved domains identified, the encoded protein has been cataloged as a transcription factor (Amselem et al., 2011; Simon et al., 2013) and, therefore, a role in regulation of gene expression might be expected. GAL4-like TFs contain a DNA binding domain and an activator domain. However, the Bcin04g03490 protein does not have such an activator domain, and instead it possesses a sugar acetyl transferase domain.

### Mutations in the Mycelial Non-pathogenic Isolates Are Allelic

**FIGURE 6:**
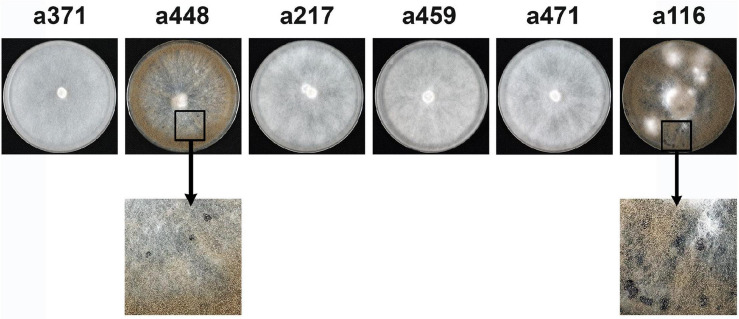
Complementation analysis between mutations in the Bcin04g03490 alleles. Primary transformants of isolate B371 generated with plasmids harboring the alleles indicated were transferred to selection plates and incubated for 14 days at 22°C in dark conditions. Sporulation and production of sclerotia were evaluated as indicators of functional complementation.

B371 was selected for genetic analysis as a representative isolate of the group of seven mycelial non-pathogenic isolates identified. They are all phenotypically similar, but this similarity does not imply that they are all altered in the same gene. To test this situation the plasmids containing the Bcin04g03490 alleles from three mycelial non-pathogenic isolates, B217, B459 and B471, together with the plasmids harboring the a371 and the a116 alleles, were transformed into B371 protoplasts. Plasmid pBAS23, which contains the a448 allele, was used as a reference control. Sporulation and formation of sclerotia were evaluated in the transformants generated as indicators of functional complementation. As shown in Figure 6, transformation with allele a448, but not with allele a371, restored the ability to sporulate and to produce sclerotia, as expected. It also restored pathogenicity (see Figure 3) and light responses (not shown). Transformations with alleles a217, a459 and a471 did not rescue these phenotypes, indicating that the mutations in these three alleles are allelic with the mutation in allele a371. The a116 allele complemented the mutation in B371 restoring sporulation and sclerotia formation (Figure 3) and pathogenicity and light responses (not shown), indicating that B116 is altered in a different gene, not in Bcin04g03490.

In order to gain information about the nature of the alterations in gene Bcin04g03490 in the B217, B459 and B471 isolates, the corresponding alleles were sequenced. The alleles from the other three mycelial non-pathogenic isolates, B286, B350 and B468, were also sequenced. In all cases, the coding region plus 300 nt from the 5′ and 3′ flanking regions were sequenced. Results are presented in Figure 5B. In comparison with the reference B05.10 isolate Bcin04g03490 allele, the alleles from all the non-pathogenic isolates shared a C(G) to T(A) transition at position + 130, which determines a E44K substitution, but it is also found in the pathogenic isolate B448. It is, therefore, a polymorphism characteristic of all the local isolates considered in this work which does not affect the phenotypes of interest. Allele a350 was found to be identical in sequence to allele a371. Isolate B371 and isolate B350 were recovered from different bunches collected from different plants in the same vineyard in the same collection date. They likely represent clones of the same genotype. The G722R substitution in the protein sequence affects a residue in the LbH_MAT_GAT domain. The other five alleles all harbor mutations in the Bcin04g03490 coding region and these are different from those found in allele a371. Three alleles, a459, a468 and a471, have mutations generating early STOP codons that result in truncated versions of the protein, 223, 142 and 359 amino acids in length, respectively. Allele a217 was shown to harbor two silent mutations, a G(C) to A(T) transition at position 678 and a A(T) to G(C) transition at position 1014. In addition, a C(G) to T(A) transition was identified, this one causing a C330Y substitution, involving one of the Cysteine residues forming part of the DNA binding domain characteristic of GAL4. Finally, allele a286 was found to harbor a silent G(C) to A(T) transition at position 678 and a A(T) to C(G) transversion at position 1947. The latter mutation determines a Y633D substitution in the LbH_MAT_GAT domain.

### Deletion of Bcin04g03490 in B05.10 Causes the B371 Phenotype

**FIGURE 7:**
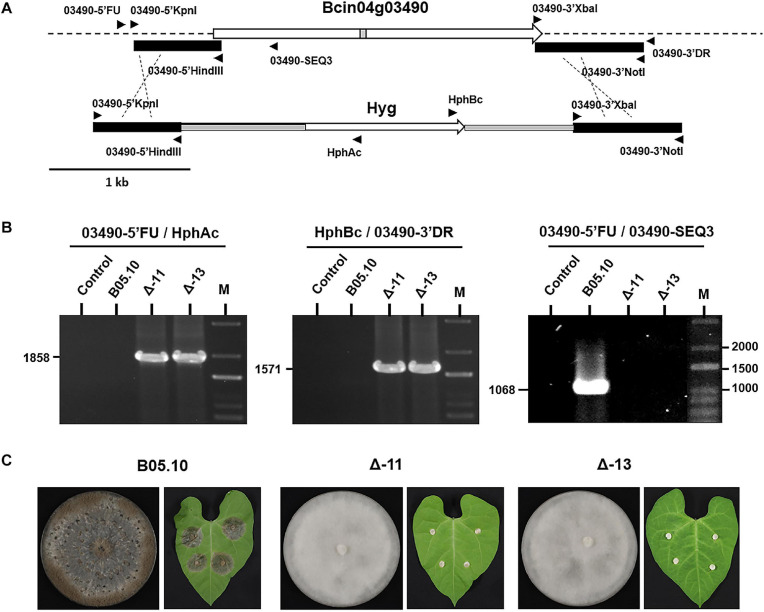
Generation of ΔBcin04g03490 mutants. **(A)** Organization of the genomic copy of Bcin04g03490 and structure of the construct used for gene replacement. The flaking sequences conserved to generate the replacement construct are indicated with black boxes. The positions and orientation of the primers used to generate the construct and to confirm the replacement are indicated. **(B)** PCR based identification of ΔBcin04g03490 mutants. In all cases reactions were carried out with water (Control), genomic DNA of B05.10 or genomic DNA of two deletion mutants as template. The sizes of the diagnostic bands are indicated. **(C)** Phenotype of the reference B05.10 isolate and of two deletion mutants during growth in MEA plates (14 days at 22°C in darkness) and in inoculations in bean leaves (images taken 72 h after inoculation).

The functions of the Bcin04g03490 gene product in development and pathogenicity has been unraveled in the genetic background of mycelial non-pathogenic field isolates. To obtain additional evidence about its role in these processes in *B. cinerea*, the Bcin04g03490 allele was deleted from the reference isolate B05.10 using gene replacement methods. The phenotype of two independent mutants was analyzed in comparison with that of the reference isolate and that of the field mycelial isolate B371. Both B05.10 knockout mutants showed the inability to sporulate, to produce sclerotia and to infect the bean leaves, the phenotype characteristic of the non-pathogenic mycelial field isolates identified in this work (Figure 7). They do not respond to light at all (not shown).

### Orthologs of Bcin04g03490

**FIGURE 8:**
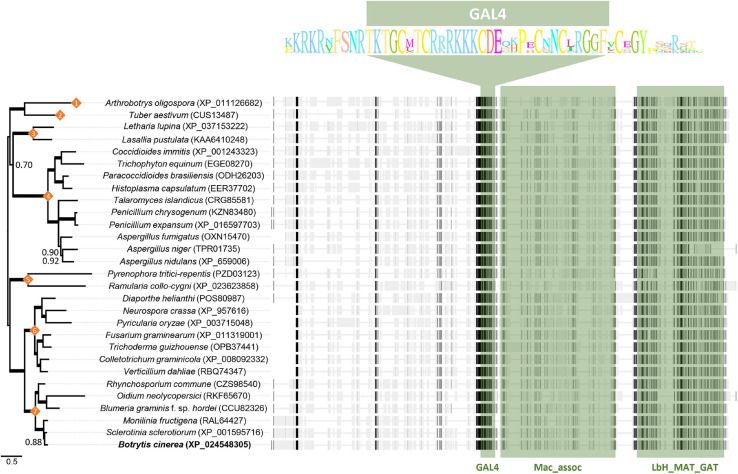
Alignment and phylogenetic tree of representative selected sequences from Pezizomycotina species detected by BLAST analysis with the Bcin04g03490 encoded protein. Numbers on nodes indicate the taxonomic class of the selected species: (1) Orbiliomycetes; (2) Pezizomycetes; (3) Lecanoromycetes; (4) Eurotiomycetes; (5) Dothideomycetes; (6) Sordariomycetes; (7) Leotiomycetes. Bayesian posterior probability (BPP) values (above 0.50) are reported next to the nodes while thicker branches indicate BPP values of 1. On the right of each species name the sequence is presented as a discontinuous thick line. Thin segments represent gaps introduced to allow optimal alignment between regions represented as thick segments in which similar residues are marked in dark gray. The regions where the GAL4, Mac_assoc and LbH_MAT_GAT domains are located are shown in green. The highly conserved residues of the GAL4-like domain in the proteins analyzed are displayed as sequence logos.

No functional characterization of the Bcin04g03490 gene has been previously described. Our results indicate that its gene product plays a key role in development and pathogenicity. In order to obtain information about its orthologs in other systems a blast analysis was performed using the entire 754 protein sequence as the query. Orthologs were identified in the fungal kingdom and their presence appeared to be restricted to the Pezizomycotina within the Ascomycetes. In this group, proteins maintaining a high overall similarity through their entire length, covering the GAL4-like domain as well as the LbM_MAT_GAT domain, were identified (Figure 8). The sequences of these two domains are highly conserved in all the species considered, representative of the different classes within the Pezizomycotina, in particular the sequence of the GAL4-like domain. In addition, numerous proteins showing homology either with the region corresponding to the GAL4-like DNA binding domain sequence in the central part of the Bcin04g03490 protein or with the LbM_MAT_GAT domain in the carboxy terminus of the protein were detected. The first group of proteins represent GAL4-like domain containing proteins which belong to members of the different major groups within the fungal kingdom, not being restricted to the Ascomycetes. The second group includes proteins with an acetyltransferase domain and are identified in members of all taxonomic groups, from archaea to plants.

In the full set of genes and proteins detected in the BLAST analysis little functional information is given. In most cases annotations derive from bioinformatic analysis and no information about the process in which a given gene participates is offered. Database searchers revealed that in the case of *N. crassa* information about mutants specifically altered in the orthologous gene, FF-7, have been presented. The gene (NCU04001) has been cataloged as a Zn2Cys6 transcription factor and mutants in the gene have been generated in the course of a large-scale functional study of transcription factors in *Neurospora* (Colot et al., 2006; Carrillo et al., 2017). The gene is not essential, and the deletion mutants form conidia on slants, but they show alterations in sexual development, being unable to produce protoperithecia, perithecia and ascospores (Carrillo et al., 2017). In *Fusarium graminearum* deletion mutants in 657 genes encoding transcription factors have been generated, among them the orthologs of the 103 TFs studied in *N. crassa* (Colot et al., 2006). In this system the ortholog of FF-7, and therefore of Bcin04g03490, identified as FGSG-10069, has been shown to be important for growth and sexual development and, in this case, also for virulence (Son et al., 2011).

## Discussion

Variation lies at the heart of genetic analysis. In this work we focused on the characterization of a collection of *B. cinerea* field isolates that showed large physiological differences among individuals regarding their ability to infect the vine. Medium aggressiveness isolates predominated, though very aggressive isolates as well as weak pathogens were also found (Acosta Morel et al., 2019). We selected a group of eight isolates that are completely non-pathogenic on grapevine for genetic analysis.

Phenotypically these isolates separate into two different types and the genetic analysis performed showed that both groups of mutants define distinct complementation groups, each altered in a different gene. Isolate B116 is the sole member of the first group, while the seven mycelial isolates form a second and homogeneous group. These mycelial isolates all harbor mutations in gene Bcin04g03490 and show identical alterations in pathogenicity and in developmental processes. The alterations include the production of aerial, sterile mycelium which does not sporulate at all, the failure to produce sclerotia and also the elimination of the capacity to sense and respond to light modifying developmental programs. Numerous fundamental processes are affected by mutations in a single gene, indicating that the Bcin04g03490 gene plays a key regulatory role in the physiology of the pathogen. Likely, the gene altered in isolate B116 will also be a key gene playing an essential role regulating development and pathogenicity, given its phenotypes. The genetic analysis of crosses between isolate B116 and the aggressive isolate B448 is currently in progress.

The annotation of the *B. cinerea* genome indicates that the Bcin04g03490 gene is cataloged as a gene encoding a transcription factor of the fungal-specific Zn(II)2Cys6 (C6) binuclear cluster class (Amselem et al., 2011; Simon et al., 2013). In all cases the criterium for this classification was the presence of a Zn(II)2Cys6 binuclear cluster DNA binding domain characterized by the highly conserved motif CX2CX6CX6CX2CX6C. The Bcin04g03490 protein has this DNA binding domain, but it is located in the central region of the protein. Moreover, the C-terminus of the encoded protein contains a predicted acetyltransferase domain. Structurally, therefore, the Bcin04g03490 protein differs from the “conventional” Zn(II)2Cys6 TFs. We propose that the Bcin04g03490 protein binds to DNA and speculate that it functions in regulating gene expression through a mechanism that involves the acetylation of specific substrates, such as proteins in the chromatin complex.

The distinct mutations in the seven Bcin04g03490 alleles all represent loss of function substitutions. Three of them have nonsense mutations that give rise to small, truncated versions of the protein and for which it is possible to predict a total loss of function. The allelic variants of the four remaining mycelial isolates result from nucleotide changes that determine amino acid substitutions. Notably, in allele a217 the nucleotide substitution affects the second Cysteine of the six Cysteines that form the Zn-binding motif characteristic of this type of domains. These six residues are absolutely conserved (Todd and Andrianopoulos, 1997) and are essential for DNA-binding (Johnston and Dover, 1987; Pfeifer et al., 1989; Parsons et al., 1992; Todd and Andrianopoulos, 1997). Mutations in alleles a371 (and a350) and a286 involved residues affecting the LbH_MAT_GAT domain. As they result in complete loss of function, those mutations identify two residues essential for this activity: the G residue at position 722 (substituted by R in the a371 protein), and the Y residue at position 633 (substituted by D in the a286 protein).

Bcin04g03490 is a gene with a major effect that regulates developmental processes and pathogenicity. Numerous studies have analyzed the role of signaling cascades and genetic regulatory factors that affect morphogenesis and virulence and demonstrate the existence of connections between differentiation and developmental processes and pathogenicity (Schumacher and Tudzynski, 2012). During the last decade, evidence has accumulated that shows a fundamental role of light as a key regulatory factor of development and virulence in *B. cinerea* (Canessa et al., 2013). It is interesting to note that the non-pathogenic mutants identified in this study show phenotypes that resemble those of the “blind” isolates. They have lost their ability to respond to light and, thereby, their ability to produce conidia and sclerotia in a light-regulated manner (Canessa et al., 2013; Schumacher, 2017). The B116 isolate displays the characteristics typical of the “always conidia” isolates, which show hyperconidiation in a light-independent manner, and do not produce sclerotia. On the other hand, the isolates of the group represented by the isolate B371 show the characteristics of the “always mycelia” isolates, producing a sterile aerial mycelium with a “fluffy” appearance and that do not produce sclerotia. The mycelial fluffy phenotype is frequently described in natural populations of the pathogen. The mycelial-type isolates described by Paul (1929) probably represent fluffy isolates. It is striking that those isolates were reported to be the most actively parasitic isolates. Following Paul’s classification, several authors have described “mycelial type” isolates (Faretra et al., 1988; Martinez et al., 2003: Korolev et al., 2008). More recently, Canessa et al. (2013) have described 4 isolates out of 72 wild strains that failed to produce any reproductive structures, among them strain J47a, always producing undifferentiated mycelia of fluffy appearance. When evaluated, the mycelial isolates were shown to be able to infect the host plant (Martinez et al., 2003; Korolev et al., 2008). This is a characteristic that our mycelial isolates do not share. A mycelial fluffy phenotype has been described in several occasions in strains generated in laboratory conditions in the course of the analysis of factors controlling development in *B. cinerea*. It was found associated with modulation of G protein signaling (Doehlemann et al., 2006) or with deletion of the bZIP TF BcATF1 (Temme et al., 2012). Deletion of the latter TF generates mutants impaired in conidia production which do not differentiate sclerotia. However, they show extremely vigorous growth in axenic culture and marked increase in colonization of different hosts. On the other hand, overexpression of both *wcl1* and *wcl2* increases formation of aerial hyphae associated with reduced conidiation, yielding colonies with a fluffy appearance (Canessa et al., 2013). None of these “natural or laboratory fluffy” isolates have exactly the phenotype shown by the non-pathogenic mycelial isolates identified in this work, which arise from the alteration of the Bcin04g03490 gene. It will be interesting to determine if the gene Bcin04g03490 is altered in the mycelial isolates previously described. It will also be of interest to evaluate the expression and function of the white collar complex genes, and of other regulatory genes participating in development and pathogenicity, in the Bcin04g03490 mutants. A global expression analysis in the mutants will be highly informative in this context.

The Bcin04g03490 gene and its orthologs appear to be specific of the Pezizomycotina within the Ascomycetes. It is not essential for the survival of the organism, but it plays an important role in controlling developmental processes. In the three systems in which it has been functionally characterized, its elimination disturbs the ability to complete the sexual cycle. *In B. cinerea* this is likely because the mutant does not produce sclerotia. In *N. crassa* and in *F. graminearum* fruiting bodies are not produced at all (Colot et al., 2006; Son et al., 2011; Carrillo et al., 2017). A general function in controlling sexual development can be proposed for the gene product. Other alterations inform about functions which appear to be species specific. Thus, in *N. crassa* radial growth and conidia production is not altered (Carrillo et al., 2017), while in *F. graminearum* radial growth is reduced (Son et al., 2011) and in *B. cinerea* sporulation is completely abolished. Finally, in the two plant pathogenic fungi the virulence is affected, in *F. graminearum* being reduced (Son et al., 2011) but completely annulled in *B. cinerea*.

In other fungal necrotrophs, mutants showing the phenotypes displayed by the mycelial non-pathogenic isolates altered in Bcin04g03490 have not been reported. The UV-induced *Sclerotinia sclerotiorum* mutant A2 (Godoy et al., 1990) resembles the *B. cinerea* mutants in some aspects, as it is non-pathogenic and does not produce sclerotia. It also fails to produce oxalic acid, an aspect which has not been evaluated in the *B. cinerea* mutants. The nature of the genetic defects in the A2 mutant has not been determined and evaluation of available evidence showed that UV-induced mutants harbored previously unrecognized genetic alterations (Xu et al., 2018). Although different mutations could have been induced in its genetic background, it might be worthwhile exploring whether the *S. sclerotiorum* ortholog of the Bcin04g03490 gene is altered in the A2 mutant.

The polymorphism responsible for the phenotypes observed between isolates B448 and B371 has been identified by means of the analysis of genetic variation between them at the genome scale. For both isolates the levels of polymorphism in relation to a reference isolate are in the range reported for other field isolates (Blanco-Ulate et al., 2013; Atwell et al., 2015, 2018; van Kan et al., 2017; Soltis et al., 2019) and support previous observations highlighting the existence of high levels of standing variation in natural populations of the pathogen (Atwell et al., 2018; Soltis et al., 2019). This holds true at the gene level. In each of the seven Bcin04g03490 alleles sequenced at least two, and up to four, SNPs were detected. Our selection criterium imposes an important bias, since only non-pathogenic mycelial isolates are being analyzed. Therefore, each allele should harbor at least one major impact polymorphism. But in the seven alleles at least one additional polymorphism, either silent or functionally not relevant, is found.

The fact that non-pathogenic mycelial isolates are found in grape bunches in the field raises several questions from the ecological perspective. How do they arise and how are they maintained? If the corresponding Bcin04g03490 mutant alleles are in the populations, even with a low frequency, it is possible that macroconidia carrying the mutation in homokaryosis are produced from heterokaryotic mycelium. It is unknown whether or not those macroconidia would germinate and develop into hyphae and infect the host tissues, but certainly the mycelium produced would display the phenotype characteristic of the natural mycelial non-pathogenic mutants purified. It would grow saprophytically, but if it does not produce spores and sclerotia and is not able to infect the plant tissues, its propagation and dispersion capacity would certainly be limited, and the fitness of the pathogen would decrease. Then, how and why are they maintained in the populations? Perhaps these isolates could be able to infect and multiply in other hosts, or even sporulate or produce sclerotia under particular environmental conditions. However, this appears to be unlikely since the mycelial isolates did not infect any host that we tested (*S. lycopersicum*, *Prunus domestica*, *Arabidopsis thaliana, P. vulgaris* and several varieties of *V. vinifera)*. Furthermore, they behave as blind mutants insensitive to light. The corresponding Bcin04g03490 mutant alleles would only be maintained in the populations through propagations of heterokaryotic mycelium. Alternatively, they could be maintained through sexual reproduction in crosses in which these isolates participate as the male parent. Their capacity to produce microconidia that act as spermatia in sexual reproduction in crosses (Faretra et al., 1988) is not compromised in the mutants, as has been shown in this work. But if they do not confer any selective advantage, their frequency would decrease over time. It is interesting to note that, although considered a typical necrotroph, *B. cinerea* has been shown to be capable of behaving as an endophyte, colonizing plants internally without causing any disease symptom (van Kan et al., 2014). Once inside the host plant, if able to penetrate, the non-pathogenic isolates would disseminate without depending on the production of conidia or of sclerotia. Moving to other plants in the field would involve long distance transmission and that would be much more limited in the absence of conidia. Perhaps transmission by means of insect vectors could take place. It might be speculated that the mutations in gene Bcin04g03490 could provide an advantage when the fungus behaves, if that occurs, as an endophyte. In any case, it is intriguing why these mycelial non-pathogenic blind mutants described in this work, all altered in gene Bcin04g03490, as well as other blind mutants previously described (Canessa et al., 2013), are maintained in the populations at high frequencies. If the mycelial isolates cannot cause disease and cannot produce spores and sclerotia, it would be fair to assume that this is an evolutionary dead-end and that such genotypes would become extinct. Our disease assays cannot exclude that the isolates are able to infect or otherwise colonize (asymptomatically) other host species or tissues that we did not test in our experiments. The observation that the mycelial phenotype is discovered multiple times in a population of limited size and that different isolates carry independent nucleotide substitutions in the same gene suggests that the isolates have a good ecological fitness. This suggests that these mycelial isolates reproduce and disperse successfully, but the mechanisms conferring their competitiveness remain to be understood.

Through the analysis of natural variation, the role of a novel major effect gene in *B. cinerea* has been unraveled. The functional characterization of Bcin04g03490 brings to light an additional layer in the complex network of regulatory circuits controlling development, light sensing and pathogenicity in *B. cinerea*. Given the phenotypes displayed by the mutants, Bcin04g03490, and/or genes under its control, can be considered as attractive targets in the context of the development of alternative control strategies.

## Data Availability Statement

The datasets presented in this study are publicly available in online repositories (SRA of NCBI). Accession numbers are: B448 Genome – SRR13700579; B371 Genome – SRR13697367; Pool aggressive progeny – SRR13703232; and Pool non-aggressive progeny – SRR13703231.

## Author Contributions

JMD-M and EPB conceived and planned the overall structure. WAM, FAF, SB, and EPB performed the experimental work. RB, MT, JvK, and EPB analyzed and elaborated sequence data. RB, JvK, JMD-M, and EPB wrote the manuscript. All authors proofread the manuscript before submission.

## Conflict of Interest

The authors declare that the research was conducted in the absence of any commercial or financial relationships that could be construed as a potential conflict of interest.

## Footnotes

1 http://fungi.ensembl.org/Botrytis_cinerea

## Supplementary Material

Supplementary Figure 1 Evaluation of aggressiveness in *V. vinifera* leaves of the B448 x B371 cross progeny. The bars indicate mean values from three independent experiments. Standard deviations are shown. Non-wounded inoculated leaves were incubated at 22°C with a 16 h photoperiod for 96 h.

Supplementary Table 1 Oligonucleotides used in this work.

## References

[B1] Acosta Morel W. Marques-Costa T. M. Santander-Gordon D. Anta Fernandez F. Zabalgogeazcoa I. Vázquez de Aldana B. R. (2019). Physiological and population genetic analysis of *Botrytis* field isolates from vineyards in Castilla y Leon, Spain. *Plant Pathol.* 68 523–536. 10.1111/ppa.12967

[B2] Amselem J. Cuomo C. A. van Kan J. A. Viaud M. Benito E. P. Couloux A. (2011). Genomic analysis of the necrotrophic fungal pathogens *Sclerotinia sclerotiorum* and *Botrytis cinerea*. *PLoS Genet.* 7:e1002230. 10.1371/journal.pgen.1002230 21876677 PMC3158057

[B3] Atwell S. Corwin J. A. Soltis N. E. Subedy A. Denby K. J. Kliebenstein D. J. (2015). Whole genome resequencing of *Botrytis cinerea* isolates identifies high levels of standing diversity. *Front. Microbiol*. 6:996. 10.3389/fmicb.2015.00996 26441923 PMC4585241

[B4] Atwell S. Corwin J. Soltis N. Kliebenstein D. (2018). Resequencing and association mapping of the generalist pathogen *Botrytis cinerea*. *bioRxiv* [Preprint] 10.1101/489799

[B5] Benito-Pescador D. Santander D. Arranz M. Díaz-Mínguez J. M. Eslava A. P. van Kan J. A. L. (2016). Bcmimp1, a *Botrytis cinerea* gene transiently expressed in planta, encodes a mitochondrial protein. *Front. Microbiol*. 7:213. 10.3389/fmicb.2016.00213 26952144 PMC4767927

[B6] Birkeland S. R. Jin N. Ozdemir A. Z. Lyons R. H. Weisman L. S. Wilson T. E. (2010). Discovery of mutations in *Saccharomyces cerevisiae* by pooled linkage analysis and whole-genome sequencing. *Genetics* 186 1127–1137. 10.1534/genetics.110.123232 20923977 PMC2998298

[B7] Blanco-Ulate B. Allen G. Powell A. L. Cantu D. (2013). Draft genome sequence of *Botrytis cinerea* BcDW1, inoculum for noble rot of grape berries. *Genome Announc.* 1:e00252-13. 10.1128/genomeA.00252-13 PMC3662820 23704180

[B8] Büttner P. Koch F. Voigt K. Quidde T. Risch S. Blaich R. (1994). Variations in ploidy among isolates of *Botrytis cinerea*: implications for genetic and molecular analyses. *Curr. Genet*. 25 445–450. 10.1007/bf00351784 8082191

[B9] Canessa P. Schumacher J. Hevia M. A. Tudzynski P. Larrondo L. F. (2013). Assessing the effects of light on differentiation and virulence of the plant pathogen *Botrytis cinerea*: characterization of the white collar complex. *PLoS One* 8:e84223. 10.1371/journal.pone.0084223 24391918 PMC3877267

[B10] Carrillo A. J. Schacht P. Cabrera I. E. Blahut J. Prudhomme L. Dietrich S. (2017). Functional profiling of transcription factor genes in *Neurospora crassa*. *G3* 7 2945–2956. 10.1534/g3.117.043331 28696922 PMC5592922

[B11] Colot H. V. Park G. Turner G. E. Ringelberg C. Crew C. M. Litvinkova L. (2006). A high-throughput gene knockout procedure for *Neurospora* reveals functions for multiple transcription factors. *Proc. Natl. Acad. Sci. U.S.A.* 103 10352–10357. 10.1073/pnas.0601456103 16801547 PMC1482798

[B12] Darriba D. Taboada G. L. Doallo R. Posada D. (2011). ProtTest 3: fast selection of best-fit models of protein evolution. *Bioinformatics* 27 1164–1165. 10.1093/bioinformatics/btr088 21335321 PMC5215816

[B13] Dean R. van Kan J. A. L. Pretorius Z. A. Hammond-Kosack K. E. di Pietro A. Spanu D. (2012). The top 10 fungal pathogens in molecular plant pathology. *Mol. Plant Pathol*. 13 414–430. 10.1111/j.1364-3703.2011.00783.x 22471698 PMC6638784

[B14] Doehlemann G. Berndt P. Hahn M. (2006). Different signalling pathways involving a Gα protein, cAMP and a MAP kinase control germination of *Botrytis cinerea* conidia. *Mol. Microbiol.* 59 821–835. 10.1111/j.1365-2958.2005.04991.x 16420354

[B15] Ehrenreich I. M. Torabi N. Jia Y. Kent J. Martis S. Shapiro J. A. (2010). Dissection of genetically complex traits with extremely large pools of yeast segregants. *Nature* 464, 1039–1042. 10.1038/nature08923 20393561 PMC2862354

[B16] Elad Y. Pertot I. Cotes Prado A. M. Stewart A. (2016). “Plant hosts of *Botrytis* spp,” in *Botrytis* – *The Fungus, the Pathogen and its Management in Agricultural Systems*, eds Fillinger S. Elad Y. (Berlin: Springer), 413–486. 10.1007/978-3-319-23371-0_20

[B17] Faretra F. Antonacci E. Pollastro S. (1988). Sexual behaviour and mating system of *Botryotinia fuckeriana*, teleomorph of *Botrytis cinerea*. *J. Gen. Microbiol.* 134 2543–2550. 10.1099/00221287-134-9-2543

[B18] Godoy G. Steadman J. R. Dickman M. B. Dam R. (1990). Use of mutants to demonstrate the role of oxalic acid in pathogenicity of *Sclerotinia sclerotiorum* on *Phaseolus vulgaris*. *Physiol. Mol. Plant Pathol.* 37 179–191. 10.1016/0885-5765(90)90010-u

[B19] Hilber U. Bodmer M. Smith F. Köller W. (1994). Biolistic transformation of conidiao f *Botryotinia fuckeliana. Curr. Genet.* 25, 124–127. 10.1007/BF00309537 8087880

[B20] Johnston M. Dover J. (1987). Mutations that inactivate a yeast transcriptional regulatory protein cluster in an evolutionarily conserved DNA binding domain. *Proc. Nat. Acad. Sci. U.S.A.* 84 2401–2405. 10.1073/pnas.84.8.2401 3550810 PMC304659

[B21] Katoh K. Standley D. M. (2013). MAFFT multiple sequence alignment software version 7: improvements in performance and usability. *Mol. Biol. Evol.* 30 772–780. 10.1093/molbev/mst010 23329690 PMC3603318

[B22] Kerssies A. Bosker-van Zessen A. I. Wagemakers C. A. M. van Kan J. A. L. (1997). Variation in pathogenicity and DNA polymorphism among *Botrytis cinerea* isolates sampled inside and outside a glasshouse. *Plant Dis.* 81 781–786. 10.1094/pdis.1997.81.7.781 30861893

[B23] Korolev N. Elad Y. Katan T. (2008). Vegetative compatibility grouping in *Botrytis cinerea* using sulphate non-utilizing mutants. *Eur. J. Plant Pathol*. 122 369–383. 10.1007/s10658-008-9301-6

[B24] Leroch M. Mernke D. Koppenhoefer D. Schneider P. Mosbach A. Doehlemann G. (2011). Living colors in the gray mold pathogen *Botrytis cinerea*: codon-optimized genes encoding green fluorescent protein and mCherry, which exhibit bright fluorescence. *Appl. Environ. Microbiol*. 77 2887–2897. 21378036 10.1128/AEM.02644-10 PMC3126427

[B25] MacPherson S. Larochelle M. Turcotte B. (2006). A fungal family of transcriptional regulators: the zinc cluster proteins. *Microbiol. Mol. Biol. Rev.* 70 583–604. 10.1128/mmbr.00015-06 16959962 PMC1594591

[B26] Magwene P. M. Willis J. H. Kelly J. K. (2011). The statistics of bulk segregant analysis using next generation sequencing. *PLoS Comput. Biol.* 7:e1002255. 10.1371/journal.pcbi.1002255 22072954 PMC3207950

[B27] Martinez F. Blancard D. Lecomte P. Levis C. Dubos B. Fermaud M. (2003). Phenotypic differences between vacuma and transposa subpopulations of *Botrytis cinerea*. *Eur. J. Plant Pathol*. 109 479–488.

[B28] Michelmore R. W. Paran I. Kesselli R. V. (1991). Identification of markers linked to disease resistance genes by bulked segregant analysis: a rapid method to detect markers in specific genomic regions using segregating populations. *Proc. Natl. Acad. Sci. U.S.A.* 88 9828–9832. 10.1073/pnas.88.21.9828 1682921 PMC52814

[B29] Pan T. Coleman J. E. (1990). The DNA binding domain of GAL4 forms a binuclear metal ion complex. *Biochemistry* 29 2023–2029. 10.1021/bi00464a019 2186803

[B30] Parsons L. M. Davis M. A. Hynes M. J. (1992). Identification of functional regions of the positively acting regulatory gene amdR from *Aspergillus nidulans*. *Mol. Microbiol*. 6 2999–3007. 10.1111/j.1365-2958.1992.tb01758.x 1479891

[B31] Parts L. Cubillos F. A. Warringer J. Jain K. Salinas F. Bumpstead S. J. (2011). Revealing the genetic structure of a trait by sequencing a population under selection. *Genome Res.* 21, 1131–1138. 10.1101/gr.116731.110 21422276 PMC3129255

[B32] Paul W. R. C. (1929). A comparative morphological and physiological study of a number of strains of *Botrytis cinerea* Pers. with special reference to their virulence. *Trans. Br. Mycol. Soc.* 14 118–135. 10.1016/s0007-1536(29)80036-8

[B33] Pedro H. Yates A. D. Kersey P. J. De Silva N. H. (2019). Collaborative annotation redefines gene sets for crucial phytopathogens. *Front. Microbiol*. 10:2477. 10.3389/fmicb.2019.02477 31787936 PMC6854995

[B34] Pfeifer K. Kim K. S. Kogan S. Guarente L. (1989). Functional dissection and sequence of yeast HAP1 activator. *Cell* 56 291–301. 10.1016/0092-8674(89)90903-3 2643482

[B35] Pomraning K. R. Smith K. M. Freitag M. (2011). Bulk segregant analysis followed by high-throughput sequencing reveals the *Neurospora* cell cycle gene, ndc-1, to be allelic with the gene for ornithine decarboxylase, spe-1. *Eukaryot. Cell* 10 724–733. 10.1128/ec.00016-11 21515825 PMC3127673

[B36] Porquier A. Morgant G. Moraga J. Dalmais B. Luyten I. Simon A. (2016). The botrydial biosynthetic gene cluster of *Botrytis cinerea* displays a bipartite genomic structure and is positively regulated by the putative Zn(II)2Cys6 transcription factor BcBot6. *Fungal Genet. Biol*. 96 33–46. 10.1016/j.fgb.2016.10.003 27721016

[B37] Raeder U. Broda P. (1985). Rapid preparation of DNA from filamentous fungi. *Lett. Appl. Microbiol*. 1 17–20. 10.1111/j.1472-765x.1985.tb01479.x

[B38] Raposo R. Delcan J. Gómez V. Melgarejo P. (1996). Distribution and fitness of isolates of *Botrytis cinerea* with multiple fungicide resistance in Spanish greenhouses. *Plant Pathol*. 45 497–505. 10.1046/j.1365-3059.1995.d01-140.x

[B39] Reis H. Pfiffi S. Hahn M. (2005). Molecular and functional characterization of a secreted lipase from *Botrytis cinerea*. *Mol. Plant Pathol.* 6, 257–267. 10.1111/j.1364-3703.2005.00280.x 20565655

[B40] Romanazzi G. Feliziani E. (2014). “*Botrytis Cinerea* (Gray Mold),” in *Postharvest Decay*, ed. Bautista-Baños S. (San Diego, CA: Academic Press), 131–146. 10.1016/B978-0-12-411552-1.00004-1

[B41] Ronquist F. Huelsenbeck J. P. (2003). MrBayes 3: bayesian phylogenetic inference under mixed models. *Bioinformatics* 19 1572–1574. 10.1093/bioinformatics/btg180 12912839

[B42] Schlötterer C. Tobler R. Kofler R. Nolte V. (2014). Sequencing pools of individuals — mining genome-wide polymorphism data without big funding. *Nat. Rev. Genet*. 15:749. 10.1038/nrg3803 25246196

[B43] Schumacher J. (2017). How light affects the life of *Botrytis*. *Fungal Genet. Biol*. 106 26–41. 10.1016/j.fgb.2017.06.002 28648816

[B44] Schumacher J. Tudzynski P. (2012). “Morphogenesis and infection in *Botrytis cinerea*,” in *Morphogenesis and Pathogenicity in Fungi*, eds Pérez-Martín J. Di Pietro A. (Berlin: Springer), 225–241. 10.1007/978-3-642-22916-9_11

[B45] Schumacher J. Pradier J. M. Simon A. Traeger S. Moraga J. Collado I. G. (2012). Natural variation in the VELVET gene bcvel1 affects virulence and light-dependent differentiation in *Botrytis cinerea*. *PLoS One* 7:e47840. 10.1371/journal.pone.0047840 23118899 PMC3485325

[B46] Shelest E. (2008). Transcription factors in fungi. *FEMS Microbiol. Lett*. 286 145–151. 10.1111/j.1574-6968.2008.01293.x 18789126

[B47] Shelest E. (2017). Transcription factors in fungi: TFome dynamics, three major families, and dual-specificity TFs. *Front. Genet.* 8:53. 10.3389/fgene.2017.00053 28523015 PMC5415576

[B48] Simon A. Dalmais B. Morgant G. Viaud M. (2013). Screening of a *Botrytis cinerea* one-hybrid library reveals a Cys2His2 transcription factor involved in the regulation of secondary metabolism gene clusters. *Fungal Genet. Biol.* 52 9–19. 10.1016/j.fgb.2013.01.006 23396263

[B49] Soltis N. E. Atwell S. Shi G. Fordyce R. Gwinner R. Gao D. (2019). Interactions of tomato and *Botrytis cinerea* genetic diversity: parsing the contributions of host differentiation, domestication, and pathogen variation. *Plant Cell* 31 502–519. 10.1105/tpc.18.00857 30647076 PMC6447006

[B50] Son H. Seo Y. S. Min K. Park A. R. Lee J. Jin J. M. (2011). A phenome-based functional analysis of transcription factors in the cereal head blight fungus, *Fusarium graminearum*. *PLoS Pathog.* 7:e1002310. 10.1371/journal.ppat.1002310 22028654 PMC3197617

[B51] Swinnen S. Schaerlaekens K. Pais T. Claesen J. Hubmann G. Yang Y. (2012). Identification of novel causative genes determining the complex trait of high ethanol tolerance in yeast using pooled-segregant whole-genome sequence analysis. *Genome Res.* 22 975–984. 10.1101/gr.131698.111 22399573 PMC3337442

[B52] Temme N. Oeser B. Massaroli M. Heller J. Simon A. González Collado I. (2012). BcAtf1, a global regulator, controls various differentiation processes and phytotoxin production in *Botrytis cinerea*. *Mol. Plant Pathol*. 13 704–718. 10.1111/j.1364-3703.2011.00778.x 22293085 PMC6638710

[B53] ten Have A. Mulder W. Visser J. van Kan J. A. (1998). The endopolygalacturonase gene Bcpg1 is required for full virulence of *Botrytis cinerea*. *Mol. Plant Microbe Interact.* 11 1009–1016. 10.1094/MPMI.1998.11.10.1009 9768518

[B54] Todd R. B. Andrianopoulos A. (1997). Evolution of a fungal regulatory gene family: the Zn(II)2Cys6 binuclear cluster DNA binding motif. *Fungal Genet. Biol*. 21 388–405. 10.1006/fgbi.1997.0993 9290251

[B55] van Kan J. A. L. (2006). Licensed to kill: the lifestyle of a necrotrophic plant pathogen. *Trends Plant Sci*. 11 247–253. 10.1016/j.tplants.2006.03.005 16616579

[B56] van Kan J. A. L. Shaw M. W. Grant-Downton R. T. (2014). Botrytis species: relentless necrotrophic thugs or endophytes gone rogue? *Mol. Plant Pathol.* 15, 957–961. 10.1111/mpp.12148 24754470 PMC6638755

[B57] van Kan J. A. L. Stassen J. H. Mosbach A. Van Der Lee T. A. Faino L. Farmer A. D. (2017). A gapless genome sequence of the fungus *Botrytis cinerea*. *Mol. Plant Pathol*. 18 75–89. 10.1111/mpp.12384 26913498 PMC6638203

[B58] Walker A.-S. Gladieux P. Decognet V. Fermaud M. Confais J. Roudet J. (2015). Population structure and temporal maintenance of the multihost fungal pathogen *Botrytis cinerea*: causes and implications for disease management. *Environ. Microbiol*. 17 1261–1274. 10.1111/1462-2920.12563 25040694

[B59] Weirauch M. T. Hughes T. R. (2011). A catalogue of eukaryotic transcription factor types, their evolutionary origin, and species distribution. *Subcell*. *Biochemistry* 52 25–73. 10.1007/978-90-481-9069-0_3 21557078

[B60] Wenger J. W. Schwartz K. Sherlock G. (2010). Bulk segregant analysis by high-throughput sequencing reveals a novel xylose utilization gene from *Saccharomyces cerevisiae*. *PLoS Genet*. 6:e1000942. 10.1371/journal.pgen.1000942 20485559 PMC2869308

[B61] Williamson B. Tudzynski B. Tudzynski P. van Kan J. A. L. (2007). *Botrytis cinerea*: the cause of grey mould disease. *Mol. Plant Pathol*. 8 561–580. 10.1111/j.1364-3703.2007.00417.x 20507522

[B62] Xu L. Li G. Jiang D. Chen W. (2018). *Sclerotinia sclerotiorum*: an evaluation of virulence theories. *Annu. Rev. Phytopathol.* 56 311–338. 10.1146/annurev-phyto-080417-050052 29958073

[B63] Zhang L. Lubbers R. J. M. Simon A. Stassen J. H. M. Ribera P. R. V. Viaud M. (2015). A novel Zn2Cys6 transcription factor BcGaaR regulates D-galacturonic acid utilization in *Botrytis cinerea*. *Mol. Microbiol.* 100 247–262. 10.1111/mmi.13314 26691528

[B64] Zhang L. Thiewes H. van Kan J. A. L. (2011). The D-galacturonic acid catabolic pathway in *Botrytis cinerea*. *Fungal Genet. Biol*. 48 990–997. 10.1016/j.fgb.2011.06.002 21683149

