# Supplementary figures and images for: A Major Effect Gene Controlling Development and Pathogenicity in Botrytis cinerea Identified Through Genetic Analysis of Natural Mycelial Non-pathogenic Isolates
Source: Front Plant Sci. 2021 Apr 14;12:663870. doi: 10.3389/fpls.2021.663870 (PMC8079791; doi:10.3389/fpls.2021.663870)

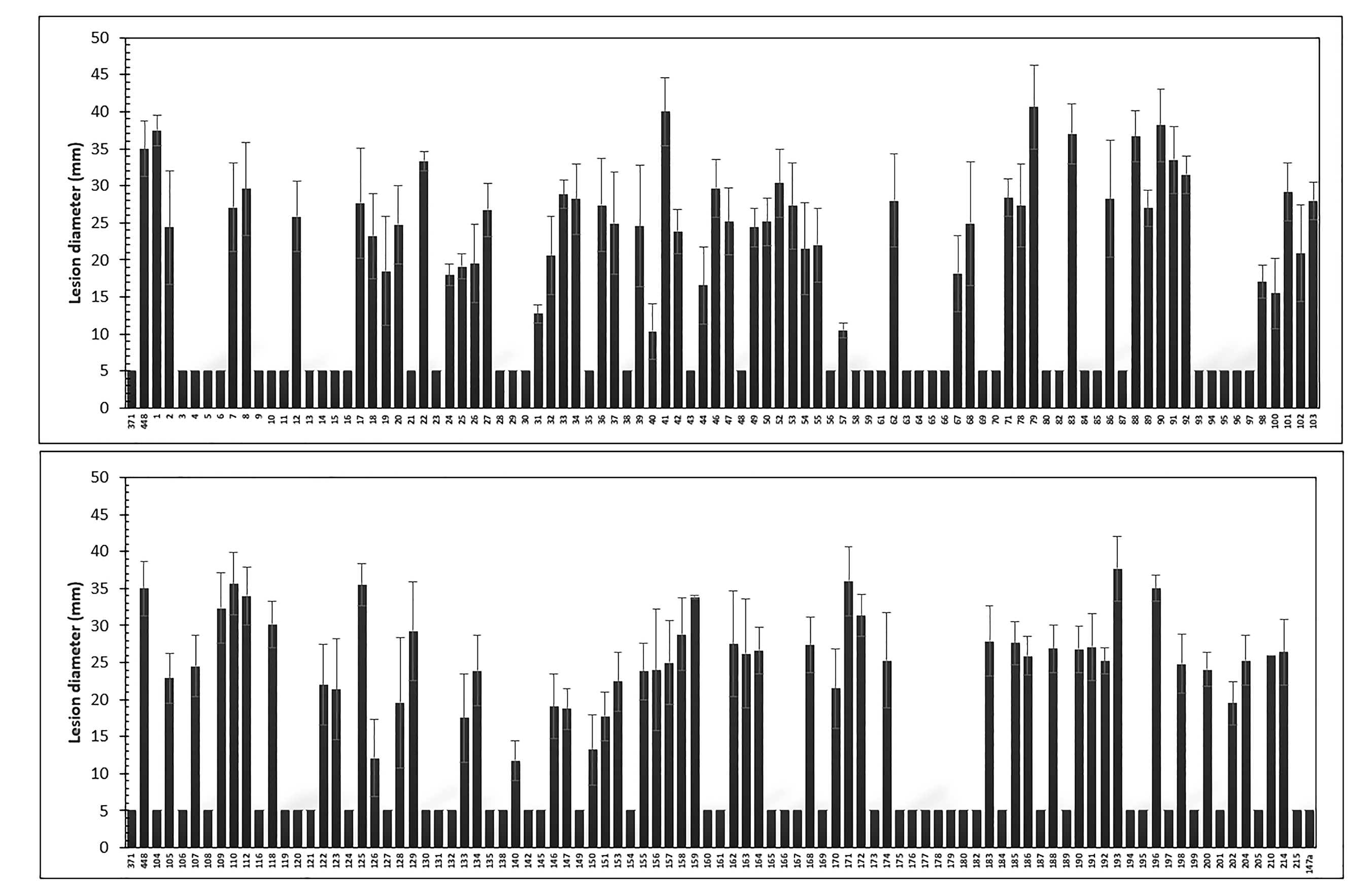

Supplement: Supplementary Figure 1 — Evaluation of aggressiveness in V. vinifera leaves of the B448 x B371 cross progeny. The bars indicate mean values from three independent experiments. Standard deviations are shown. Non-wounded inoculated leaves were incubated at 22°C with a 16 h photoperiod for 96 h. [file Image_1.TIF]
