# Supplementary material for: A Major Effect Gene Controlling Development and Pathogenicity in Botrytis cinerea Identified Through Genetic Analysis of Natural Mycelial Non-pathogenic Isolates
Source: Front Plant Sci. 2021 Apr 14;12:663870. doi: 10.3389/fpls.2021.663870 (PMC8079791; doi:10.3389/fpls.2021.663870)
Supplement: Supplementary Table 1 — Oligonucleotides used in this work. [file Table_1.DOCX]

*Table S1. Oligonucleotides used in this work. Sequences underlined correspond to the attB sites incorporated in the primers to generate the PCR products to be used as substrates in the Gateway BP recombination reactions. Nucleotides in bold and underlined indicate the substitutions introduced to generate the corresponding restriction sites.*

| **Oligonucleotide** | **Sequence 5’-3’** | **Description** |
| --- | --- | --- |
| 4S5-F | GGGGACAAGTTTGTACAAAAAAGCAGGCTCTCGGATTTGGGGGGTTGATG | Design of pBAS1 |
| 4S5-R | GGGGACCACTTTGTACAAGAAAGCTGGGTCGACTAATCACCCTTTGGGA |  |
| 4S6-F | GGGGACAAGTTTGTACAAAAAAGCAGGCTAAGTTGAAGAAAGAGGGGGG | Design of pBAS2 |
| 4S6-R | GGGGACCACTTTGTACAAGAAAGCTGGGTCGCTATAGCTAGCTACCTAG |  |
| 4S7-F | GGGGACAAGTTTGTACAAAAAAGCAGGCTGCTGAGAAGACCAAGTCATA | Design of pBAS3 |
| 4S7-R | GGGGACCACTTTGTACAAGAAAGCTGGGTGTGGACGAATGAATGTAGTATGAG |  |
| 4S8-F | GGGGACAAGTTTGTACAAAAAAGCAGGCTGACCCAAAAGATGGCCCAAA | Design of pBAS4 |
| 4S8-R | GGGGACCACTTTGTACAAGAAAGCTGGGTTAAATACCTGGGGAAAGGCG |  |
| 4S9-F | GGGGACAAGTTTGTACAAAAAAGCAGGCTTTCGCCTTTCCCCAGGTATT | Design of pBAS5 |
| 4S9-R2 | GGGGACCACTTTGTACAAGAAAGCTGGGTCCAGTGCTCTCCGATTTGAA |  |
| 4S10-F | GGGGACAAGTTTGTACAAAAAAGCAGGCTGCAGAACTTCACACAGTCAG | Design of pBAS6 |
| 4S10-R | GGGGACCACTTTGTACAAGAAAGCTGGGTGTTGCATGTCTGTCTTAGCG |  |
| 4S11-F | GGGGACAAGTTTGTACAAAAAAGCAGGCTGCAAACAGGGCTATCTACTC | Design of pBAS7 |
| 4S11-R | GGGGACCACTTTGTACAAGAAAGCTGGGTCACATGCGTCCGTGTTATCA |  |
| 4S12-F | GGGGACAAGTTTGTACAAAAAAGCAGGCTCCCGATGCGGTTTTTATCGA | Design of pBAS8 |
| 4S12-R | GGGGACCACTTTGTACAAGAAAGCTGGGTGATATAGGTGCATTCACGGG |  |
| 4S13-F | GGGGACAAGTTTGTACAAAAAAGCAGGCTCCCGTGAATGCACCTATATC | Design of pBAS9 |
| 4S13-R | GGGGACCACTTTGTACAAGAAAGCTGGGTTTTCCCTGTCTCTCCAAAGC |  |
| 4S14-F | GGGGACAAGTTTGTACAAAAAAGCAGGCTGGTAATCATTTGACCCGTGG | Design of pBAS10 |
| 4S14-R | GGGGACCACTTTGTACAAGAAAGCTGGGTTGCCAAATTCTGCCGTGGTT |  |
| 4S15-F | GGGGACAAGTTTGTACAAAAAAGCAGGCTGCTCATGTTCGAGAAGCTCT | Design of pBAS11 |
| 4S15-R | GGGGACCACTTTGTACAAGAAAGCTGGGTCCGCTATATACCGCATCTTC |  |
| 4S16-F | GGGGACAAGTTTGTACAAAAAAGCAGGCTGGAAGATGCGGTATATAGCG | Design of pBAS12 |
| 4S16-R | GGGGACCACTTTGTACAAGAAAGCTGGGTCCAGTGGTATTGGGATCCTA |  |
| 4S17-F | GGGGACAAGTTTGTACAAAAAAGCAGGCTTGCTAATGCGCAAGGAACCA | Design of pBAS13 |
| 4S17-R | GGGGACCACTTTGTACAAGAAAGCTGGGTATCATGGGCATATACCGCGA |  |
| 4S18-F | GGGGACAAGTTTGTACAAAAAAGCAGGCTGGAGGCGCTGAATATAGAGA | Design of pBAS14 |
| 4S18-R | GGGGACCACTTTGTACAAGAAAGCTGGGTCGTATAGGAACTCTTCCCGA |  |
| 4S19-F | GGGGACAAGTTTGTACAAAAAAGCAGGCTTTTATTATAGATACCCAGCC | Design of pBAS15 |
| 4S19-R | GGGGACCACTTTGTACAAGAAAGCTGGGTAAGTAGACTTGCCGGATGTG |  |
| 4S20-F | GGGGACAAGTTTGTACAAAAAAGCAGGCTAGCCCACGATTTTAGTCTTTCAC | Design of pBAS16 |
| 4S20-R | GGGGACCACTTTGTACAAGAAAGCTGGGTTCAGGATACACCAAACCTAGTTTATTG |  |
| 4S21-F | GGGGACAAGTTTGTACAAAAAAGCAGGCTTAGGTTTGGTGTATCCTGAG | Design of pBAS17 |
| 4S21-R | GGGGACCACTTTGTACAAGAAAGCTGGGTAATGTATGTAGTATAGCTCC |  |
| 4S22-F | GGGGACAAGTTTGTACAAAAAAGCAGGCTGGCCAGTATGTCTGGAGATG | Design of pBAS18 |
| 4S22-R | GGGGACCACTTTGTACAAGAAAGCTGGGTTTGTATCTTGAGACTAGAAG |  |
| 4S23-F | GGGGACAAGTTTGTACAAAAAAGCAGGCTCTAGTCTCAAGATACAAGAG | Design of pBAS19 |
| 4S23-R | GGGGACCACTTTGTACAAGAAAGCTGGGTTTCGGTCTTGGCTCTTAGCC |  |
| 4S24-F | GGGGACAAGTTTGTACAAAAAAGCAGGCTAGAGGCTAAGAGCCAAGACC | Design of pBAS20 |
| 4S24-R | GGGGACCACTTTGTACAAGAAAGCTGGGTACGAATAATCCGAGACATCG |  |
| 4S25-F | GGGGACAAGTTTGTACAAAAAAGCAGGCTACGATGTCTCGGATTATTCG | Design of pBAS21 |
| 4S25-R | GGGGACCACTTTGTACAAGAAAGCTGGGTGCTGCCTATGCTAGGAAGGG |  |
| 4S26-F | GGGGACAAGTTTGTACAAAAAAGCAGGCTGCACGGTGATCACTTAGAGG | Design of pBAS22 |
| 4S26-R | GGGGACCACTTTGTACAAGAAAGCTGGGTTCAATTTCTTGACATCAGCC |  |
| 4S7-F-1 | GGGGACAAGTTTGTACAAAAAAGCAGGCTTGATTCGGATGGAGATCACG | Design of pBAS23 |
| 4S7-R-1 | GGGGACCACTTTGTACAAGAAAGCTGGGTGTGTATCAAGAACCTAGCCG | Design of pBAS24 |
| 03490-5’KpnI | CATCGTATACTTGGGTACCACACC | Gene Replacement |
| 03490-5’HindIII | TACAAGCT**C**CTGAGTTTCAAGAGG | Gene Replacement |
| 03490-3’XbaI | CCG**G**C**A**AGAGTTATTCGTGGTCTG | Gene Replacement |
| 03490-3’NotI | CACAACAA**G**C**GG**CC**G**CGATTCTTAATCCCACCAATC | Gene Replacement |
| 03490-SEQ1 | CTGCTATGAGGACTATCGGG | Sequencing Bcin04g03490 |
| 03490-SEQ2 | CTGGCCTGGATTCATCGCAC | Sequencing Bcin04g03490 |
| 03490-SEQ3 | GGCGATCAGGTAGATGAGTACG | Sequencing Bcin04g03490 |
| 03490-QF1 | GATTGTTGGATTGGTGGCGG | Sequencing Bcin04g03490 |
| 03490-QR1 | TCATGTGGCGGATACAGACC | Sequencing Bcin04g03490 |
| 03490-5UF | CAGTCAACAACAATCCGTGGTG | GR Diagnosis |
| 03490-3DR | AGTCAAGTCAAAAGCCAACGGC | GR Diagnosis |
| HphAc | CGGGCAGTTCGGTTTCAGGC | GR Diagnosis |
| HphBc | CGTCTGGACCGATGGCTGTG | GR Diagnosis |
